# Effects of Metabolites, Sex, Sire, and Muscle Type on Chilled Lamb Meat Colour

**DOI:** 10.3390/foods12214031

**Published:** 2023-11-05

**Authors:** Renyu Zhang, Guojie Wu, Maryann Staincliffe, John C. McEwan, Mustafa M. Farouk

**Affiliations:** 1 Food Technology & Processing Team, AgResearch Ltd., Palmerston North 4474, New Zealand; renyu.zhang@agresearch.co.nz (R.Z.); guojiewu@hotmail.com (G.W.); 2 Statistics Team, AgResearch Ltd., Hamilton 3214, New Zealand; maryann.staincliffe@agresearch.co.nz; 3 Animal Genomics Team, AgResearch Ltd., Puddle Alley, Mosgiel 9092, New Zealand; john.mcewan@agresearch.co.nz

**Keywords:** colour, glutamate, lamb, methionine, NADH, reducing waste, sex, sire, sustainability, testosterone

## Abstract

Meat is an important source of high-value protein providing sustainable nutrition for human health. The discolouration of meat results in significant waste, which threatens the sustainability of meat production in terms of availability, affordability, and utilisation. Advancing the knowledge of factors and underlying mechanisms for meat discolouration supports the sustainability transformation of meat production practices. Previous studies found that colour stability may be associated with signature changes in certain metabolites, including NADH, glutamate, methionine, and testosterone. This study aimed to confirm the effect of these metabolites and sex, sire, and muscle type on lamb meat colour. NADH and glutamate improved colour stability as evidenced by the increased metmyoglobin reductase activity, while methionine and testosterone had detrimental effects. Overall, lamb meat was discoloured with retail display for up to 10 days at 4 °C. The *semitendinosus* muscle had higher L*, b*, and hue angle and lower a* (*p* < 0.05) than other muscles, especially in ewes. Lamb meat from rams had a higher L* and hue angle and lower a* than the ewes (*p* < 0.05), especially in the colour-labile group, suggesting an interaction between sex and sire. The outcomes of this study will help make the production of meat more sustainable by assisting the meat industry in improving the selection of animals for meat production and processing practices to reduce meat waste due to discolouration.

## 1. Introduction

Colour is one of the most important attributes affecting consumers’ purchasing decision of fresh meat at retail outlets [1]. The most simple and robust predictor of meat colour acceptability by consumers is the redness (a*) of meat measured with a colourimeter [2]. A survey of 2796 individuals from across countries revealed that colour is considered “Important to very important” in consumers’ meat-purchasing decisions [2]. Consumers preferred a meat product with brighter red meat colour (higher a* and chrome) and were willing to pay USD 0.35/kg for each level of improvement to the preferred colour [3]. However, the discolouration (browning) of fresh meat during retail display is a common issue resulting in significant financial losses and meat wastage as consumers associate the brown colour with spoiled meat or poor quality [4]. A recent study by Ramanathan et al. [5] indicated that about 2.55% of beef (worth USD 3.77 billion) in the US was discarded annually due to discolouration. Meat is a highly nutritious protein source for the sustainable nutrition of human beings. Meat wastage due to discolouration is one of the limiting factors for the sustainable transformation of the meat industry. Thus, it is important to improve the understanding of the factors and underlying mechanisms influencing the properties and stability of meat colour, to reduce meat wastage and improve the sustainability of meat production and consumption.

Changes in colour properties and stability during storage have been associated with several primary factors, including intrinsic factors, such as genotype, sex, age, and breed [6,7], and extrinsic factors such as production system, post-mortem processing, and storage [8,9]. Mortimer et al. [10] reported that meat quality traits were generally of moderate heritability and suggested the potential to improve meat quality through the manipulation of genetic and phenotypic factors. Previous studies indicated meat from certain lamb sires had brighter red colour and lower discolouration rate during simulated retail display (called colour-stable sires) compared with other sires termed colour-labile sires [11]. A metabolomic comparison of lamb samples from the two sire groups (colour-stable and colour-labile sires) indicated that several metabolites differed significantly between the two groups, which may have a direct/indirect influence on their colour properties and stability [12]. A significantly higher concentration of metabolites, including nicotinamide adenine dinucleotide hydrogen (NADH), L-glutamate, malic acid, and guanosine, was detected in samples from colour-stable sires, while methionine, testosterone, and their derivatives were more abundant in colour-labile sires [12]. A follow-up study by Samuelsson et al. [13] using proteomics and metabolomics suggested the improved colour stability of lamb meat from stable sires during post-mortem storage may be associated with the improved supplementation of adenosine triphosphate (ATP) and NADH for supporting the metabolism of cells.

Thus, this study aimed to perform five experiments to first confirm the impacts of four metabolites (NADH, glutamate, methionine, and testosterone) on the colour stability of lamb meat using in vitro followed by in vivo methods; then, based on the confirmation of these impacts, we sought to further determine the effects of sex, sire, and muscle type (loin and chop muscles) on colour properties and stability of lamb meat.

## 2. Materials and Methods

### 2.1. Trial Design

**Figure 1:**
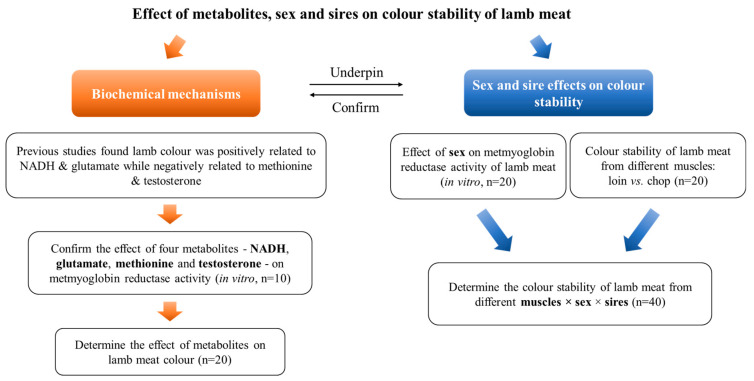
Schematic illustration of the experimental design in the current study.

The experimental design of this study is illustrated in Figure 1. We first determined the effect of four metabolites (NADH, glutamate, methionine, and testosterone) on metmyoglobin reductase activity and the colour stability of lamb meat to underpin the possible mechanisms of sex and sire effects on lamb meat colour. The changes in colour properties and stability of lamb meat from different sexes and sires during retail display were measured to confirm the current mechanisms.

### 2.2. Effect of Metabolites on Metmyoglobin Reductase Activity and Colour Stability of Lamb Meat

#### 2.2.1. Metmyoglobin Extraction and Reductase Activity

Fresh lamb loins (*m. longissimus lumborum*, *n* = 10) were purchased from a local butcher (specification No. 3434 boneless loin, fabricated according to the New Zealand meat specification guide [14]) and used to generate metmyoglobin for the measurement of metmyoglobin reductase activity. The vacuum-packaged loins were placed in a chilly bin with ice packs and transferred to the meat science laboratory within two hours. The loins were placed on a foam tray, overwrapped with oxygen-permeable film, and held in a custom-built walk-in chiller at 10 ± 2 °C for two days for the generation of metmyoglobin. Lean muscles were obtained from all ten loins (fat and connective tissues removed) and minced to prepare a pooled loin sample for extracting metmyoglobin. The extraction and purification of metmyoglobin were performed on the pooled sample to evaluate the effect of the four metabolites (glutamate, methionine, NADH, and testosterone) on the metmyoglobin reductase activity of lamb loin.

##### Chemicals

NADH (disodium salt, Lot 4Q00251, 97%, AppliChem, Darmstadt, Hessen, Germany); L-glutamate (G5667) and L-methionine (M2893) from Sigma-Aldrich, Saint Louis, MO, USA; testosterone (T1500, Lot 65F-0054, Sigma-Aldrich, Saint Louis, MO, USA); Na_2_HPO_4_ and NaH_2_PO_4_ (Scharlau Chem, Spain); Celite^®^ and K_3_Fe(CN)_6_ (Sigma-Aldrich, Saint Louis, MO, USA); K_4_Fe(CN)_6_-3H_2_O (Anala R grade, BDH Chemical Ltd., Poole, Dorset, UK); and EDTA (E4884, 98%, Sigma-Aldrich, Saint Louis, MO, USA) were used. Water was Milli-Q grade.

##### Extraction of Metmyoglobin Substrate

Metmyoglobin was extracted and purified according to Mikkelsen et al. [15], with some modifications. Briefly, 500 g of the pooled loin samples were homogenised with 1000 mL of cold water (4–7 °C) in a Waring Blender at high speed for 1 min (20 s for 3 times). The homogenate was adjusted to pH 7.5 with aqueous 2 M NH_3_ and centrifuged at 13,000× *g* for 25 min (4 °C). The supernatant was brought to 70% saturation (to remove haemoglobin) with solid ammonium sulphate and adjusted the pH to 7.5 and then spun again under the same conditions. Solid ammonium sulphate was added to the supernatant to achieve 100% saturation, followed by adjusting the pH to 7.5 to precipitate oxymyoglobin–myoglobin. Solid Celite^®^ was added into the solution (1 g per 100 mL solution) to assist with myoglobin precipitation. This solution was stirred for 30 min and then centrifuged at 20,000× *g*, 4 °C for 30 min. The filtrate was discarded. The precipitate containing oxymyoglobin–myoglobin–Celite^®^ pad was suspended in 600 mL Milli-Q water and centrifuged again at 20,000× *g*, 4 °C for 30 min to obtain the supernatant; this step was repeated twice with 200 mL Milli-Q water. The supernatants from the three purification cycles were combined and centrifuged at 20,000× *g*, 4 °C for 20 min to ensure the remaining Celite^®^ was removed. The final supernatant was oxidised with a slight excess of K_3_Fe(CN)_6_, dialysed through 14,000 MM cut-off tubing first against cold water (16 L) for 24 hrs, and then equilibrated with two cycles of 15 L phosphate buffer (2 mM, pH 7.0); each cycle was performed for 24 hrs. The resulting dialysed solution mainly containing metmyoglobin was concentrated to a final volume of 60 mL using10 kDa filtration devices (Macrosep Advance Centrifugal Device, 10 MWCO, Pall Corporation) to obtain a concentration of 0.83 mM based on spectrophotometric measurement at 525 nm (Ɛ525 = 7700 L·mol^−1^·cm^−1^) [16]. This metmyoglobin solution was split into 2 mL Eppendorf tubes and stored in a −80 °C freezer until use. Before measuring metmyoglobin reductase activity, the metmyoglobin extract was thawed in a chiller at 4 °C and centrifuged for 5 min at 10,000× *g*, and the concentration was adjusted to 0.75 mM with 2.0 mM phosphate buffer (pH 7.0).

##### Metmyoglobin Reductase Activity Assay

The metmyoglobin reductase activity of the metabolites (NADH, glutamate, methionine, and testosterone) was measured using the method by Mikkelsen, Juncher, and Skibsted [15]. Briefly, the standard assay mixture (pH 6.4) consisted of 0.10 mL of 5.0 mM EDTA, 0.10 mL of 50 mM phosphate buffer (pH 7.0), 0.10 mL of 3 mM K_4_Fe(CN)_6_, 0.20 mL of 0.75 mM metmyoglobin (MbFe(III)) in 2.0 mM phosphate buffer (pH 7.0), and 0.4 mL of 2 mM phosphate buffer (pH 7.0) containing a series of concentrations (0, 0.125, 0.25, 0.5, 1.0, 2.0, 4.0, and 8.0 mM) of these metabolites with exception of testosterone. Testosterone is insoluble in a normal aqueous solution; thus, phosphate buffer was replaced with 45% hydroxypropyl-B-cyclodextrin (Sigma-Aldrich, Saint Louis, MO, USA), and the same procedures as the other metabolites were followed. The standard assay mixture (except for metabolites) was used as the blank.

The cuvettes were placed in a thermostat-controlled spectrophotometer (Multiskan^TM^ Go, Thermo Scientific^TM^, Franklin, MA, USA). The cuvette cell temperature was set at 30 °C, and the mixture was incubated for 10 min. The reaction was initiated with the addition of 0.1 mL of 2 mM NADH into the sample cuvette. The wavelength was 580 nm (the wavelength at which the difference in absorbance between oxymyoglobin and metmyoglobin is maximal). The spectrophotometer was programmed with the kinetic model to measure and record absorbance every 2 s, up to 60 min. Metmyoglobin reductase activity was calculated as nmoles of metmyoglobin reduced (equal to nanomole of oxymyoglobin formed) per minute per gram of metabolites using a difference in molar absorptivity of 12,000 L·mol^−1^·cm^−1^ at 580 nm, during the initial linear phase of the assay.

#### 2.2.2. Colour stability of Lamb Meat Treated with Four Metabolites

Ten pairs of lamb loins (*m. longissimus lumborum*, *n* = 20) were collected from both the left and right sides of ten carcasses (Ram, approx. 11 months) on the day of slaughter. Lambs were from the same mob and slaughtered in an export-licenced meat-processing plant in New Zealand. The loins (specification No. 3434 boneless loin) were fabricated according to the New Zealand meat specification guide [14]. All the lamb samples collected in this study had normal pH values (5.60–5.80 upon 24 h post-mortem).

The lamb samples were vacuum-packaged and transferred in a refrigerated truck at (−1.5 °C) to the meat science laboratory within 2 days. Each loin was cut into five equal pieces and randomly assigned to five solutions comprising four metabolites (glutamate, methionine, NADH, and testosterone, 4 mM) and Milli-Q water as a control. The loin portion was dipped into one of five solutions with a tweezer for 3 h and then laid out on a foam tray and wrapped with PVC film to simulate the conditions for retail display. The colour stability of lamb treated with four metabolites was determined by measuring the changes in colour properties and the formation of metmyoglobin on the meat surface.

##### Colour Change Measured with a Chroma Meter

Following 0, 1, 2, 4, and 7 days under retail display light (3000 kelvin) in a custom-built walk-in chiller at 4 ± 1 °C, the surface colour of loin pieces was measured using a Minolta Chroma Meter (CR-400, Konica Minolta Inc., Tokyo, Japan) calibrated with a standard white tile prior to the measurement. Lightness, redness, and yellowness (CIE L*, a*, and b*, respectively) were measured (Illuminant D65, 8 mm diameter aperture, 10 standard observers) using the PVC film. Chroma and hue (angle) were calculated using the following equations:

$$Chroma={\left({\left(a*\right)}^{2}+{\left(b*\right)}^{2}\right)}^{1/2}$$

$$Hue=arctangent(\frac{b*}{a*})(a*>0)$$

##### Change in Spectral Reflectance

Following 7 days of retail display, the surface of loin pieces was scanned using a HunterLab MiniScan^TM^ XE Plus (HunterLabs, Reston, VA, USA) between 400 and 700 nm to obtain the spectral reflectance data to estimate the metmyoglobin formation on the muscle surface [17]. The ratio of the reflectance at 572 and 525 nm after K/S transformation was used to determine the formation of metmyoglobin as described by Hunt et al. [18] and King et al. [19]. The colour meter was calibrated using a black and white tile supplied by the manufacturer.

### 2.3. Effect of Sexes and Sires on the Colour Stability of Lamb Meat from Different Muscles

#### 2.3.1. Metmyoglobin Reductase Activity of Lamb Meat from Different Sexes

Twenty lamb loins (*m. longissimus lumborum*, *n* = 20) were collected from ten ewe and ten ram lamb carcasses on the day of slaughter. The loins (specification No. 3434 boneless loin) were fabricated according to the New Zealand meat specification guide [14]. All the loin samples were vacuum-packaged and transferred in a refrigerated truck (maintained at −1.5 °C) to the meat science laboratory within 72 h post-mortem. Whole loins (fat and connective tissue removed) from female or male lamb were minced to prepare two pooled samples of male and female lamb loins. Subsamples (500 g) were taken from these two pooled loin samples to determine the effect of sex on the metmyoglobin reductase activity. Metmyoglobin from the female or male lamb was extracted following the procedures described above (Section Extraction of Metmyoglobin Substrate). The metmyoglobin extracts were used to compare the effects of sex on colour stability by measuring the kinetics of the reduction in metmyoglobin as described above (Section Metmyoglobin Reductase Activity Assay). The kinetics of the reduction in metmyoglobin was recorded, and photographs of the colour at different reaction times were taken.

#### 2.3.2. Surface Colour Stability of Lamb from the Loin and Leg Muscles

Ten ram and ten ewe lambs (approx. 46 weeks) were obtained from two farms and fabricated under commercial conditions at an export abattoir. Upon 24 h post-mortem, loins (*n* = 20) and hindlegs (*n* = 20) were collected from the twenty lamb carcasses and vacuum-packed in barrier bags, and then transferred in a refrigerated truck at −1.5 °C to the meat science laboratory. The loin (specification No. 3434 boneless loin) and hindleg (specification No. 4001 long leg bone-in) were fabricated according to the New Zealand meat specification guide [14]. All lamb samples had normal pH (5.64 to 5.80 with a mean value of 5.72 ± 0.05), and similar pH values were observed between muscles and sexes (*p* > 0.05).

Each loin was cut into five pieces (replicates), laid out on the form tray, and overwrapped with PVC film, as described in Section 2.2.2. After removing the shank, two chops (2 cm) were taken from each leg (*n* = 10) and prepared for retail display. Changes in surface colour during the simulated retail display at 4 °C for 1, 2, 4, and 7 days were measured on loin and leg muscles (*m. semimembranosus* (SM), *m. biceps femoris* (BF), and *m. semitendinosus* (ST)) using a calibrated Minolta Chroma Meter (CR-400, Konica Minolta Inc., Tokyo, Japan). The surface colour was measured from three random positions of each loin piece or leg muscle and averaged.

#### 2.3.3. Surface Colour Stability of Lamb Chops from Different Sexes and Sires

Twenty pairs of lamb hindlegs (*n* = 40 total, specification No. 4001 long leg bone-in [14]) were collected from ten ewe and ram lamb carcasses. All lambs were from the same experimental farm (AgResearch Invermay, animal ethics approval numbers: 14369 and 14314) and fabricated in the same way as described in Section 2.3.2. Animals of different sexes were from two sires (either *m. longissimus dorsi* colour-stable or colour-labile as determined via their earlier progeny test results [20]) to give four combinations: male–stable (MS), male–labile (ML), female–stable (FS) and female–labile (FL). Lamb hindlegs were collected at 24 h post-mortem, vacuum-packaged, and transferred in a refrigerated truck (−1.5 °C) to the meat science laboratory for further analyses. All lamb samples had normal pH (5.58 to 5.85 with a mean value of 5.66 ± 0.06). A 2 cm chop was taken from each leg (*n* = 40), placed on a foam tray, and overwrapped with PVC film. Packaged lamb chops were stored in a custom-built walk-in chiller (4 °C) for 0, 1, 2, 4, 7, and 10 days. Changes in the colour properties and formation of metmyoglobin on the surface of three muscles (SM, BF, and ST) were measured on each display day.

### 2.4. Statistical Analysis

All analyses were performed in R software (version 3.4.3), using a linear mixed-effect model with the package “lme4”. Means, a least significant difference (LSD), and pairwise comparisons were performed using the “predictmeans” package. The pairwise significant differences were determined using Student’s *t*-test to separate the means at *p* < 0.05. For comparing the effect of metabolites, the interaction between retail display days as a factor was considered the fixed effect, and animal was considered the random effect. For determining the effect of muscles, sexes, and sires, the interactions between display days as a factor and muscles, sexes, and sires were included as fixed effects and days within the animal as a random effect.

## 3. Results

### 3.1. Metmyoglobin Reductase Activity

#### 3.1.1. Effect of the Four Metabolites

NADH had a positive impact on metmyoglobin reductase activity, while glutamate, methionine, and testosterone alone showed no effect (Table 1). The reduction in metmyoglobin using NADH was improved by adding glutamate (9.1%), whereas a negative impact was seen by adding methionine (−6.1%) and testosterone (−60.5%).

**Figure 2:**
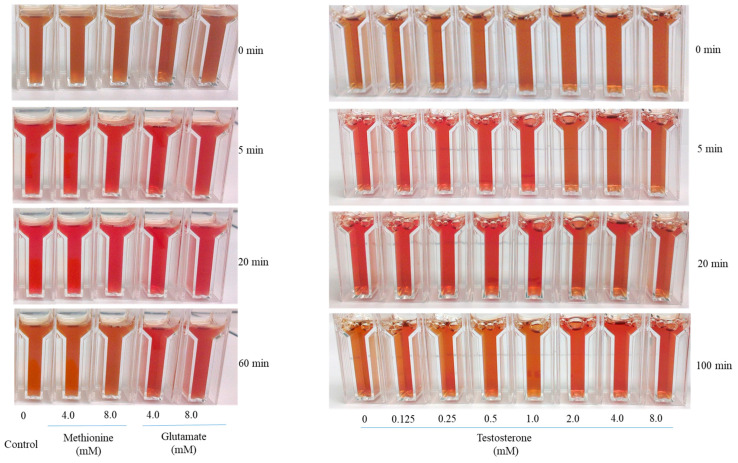
Effects of metabolites (methionine, glutamate, and testosterone) on metmyoglobin reductase activity and colour of lamb. All reaction tubes contain NADH to initiate the reaction. Tubes with NADH alone served as controls.

As shown in Figure 2, the addition of NADH to the reaction mixture initiated the reduction in metmyoglobin (brown colour, 0 min); the reaction mixture rapidly turned red until it reached to brightest red colour within 20 min and then gradually turned brown, as seen at 60 min. There was no clear difference in the reduction in metmyoglobin between the four metabolites at low concentrations between 0.1 and 1.0 mM. The impact of metabolites on metmyoglobin reductase activity differed with the increasing metabolite concentration in the reaction mixture. A higher level of methionine (4.0 and 8.0 mM) resulted in discolouration as seen at 60 min, while the increase in glutamate to 8.0 mM had no detrimental effects on colour stability (Figure 2). The effect of testosterone on the metmyoglobin reductase activity was inconsistent. The reduction rate of metmyoglobin decreased with the increasing levels of testosterone (2.0–8.0 mM) as evidenced by a delayed colour change from brown (oxidised) to bright red (reduced), which indicated a negative impact of testosterone on colour stability (Figure 2).

#### 3.1.2. Effect of Sex

**Figure 3:**
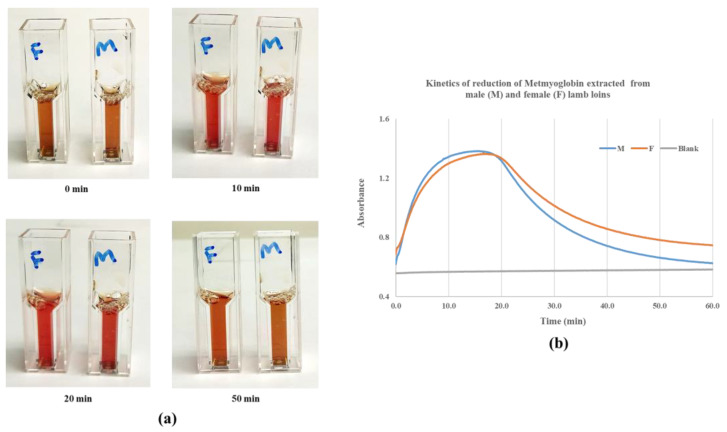
Effect of sex on metmyoglobin reductase activity: (**a**) photos of kinetic reduction in metmyoglobin extracts from males and females with NADH for 0, 10, 20, and 50 min; (**b**) kinetic changes in the absorbance of metmyoglobin extracts from males and females measured with the spectrophotometer during the reduction reaction for up to 60 min. M = male, F = female.

There was no difference in the metmyoglobin reduction (brown to red colour) between lamb meat from male and female animals using NADH within the first 20 min (Figure 3a). Lamb meat from males became browner than those of females after 50 min of reaction. This phenomenon was further supported by the kinetics of metmyoglobin reduction, as shown in Figure 3b, according to which higher absorbance (oxymyoglobin) was observed in the female animal after 20 min reaction, suggesting that the sex of the animal had an impact on metmyoglobin reductase activity and the colour stability of lamb meat.

### 3.2. Effects of Metabolites on Colour Stability of Lamb Meat

There was no significant difference observed in the instrumental colour (CIE L*, a*, b*, chroma, and hue) between any of the treatments.

The ratio of muscle surface reflectance at 572 nm to the reflectance at 525 nm determines the amount of metmyoglobin formation on the surface of a muscle with a higher (K/S572)/(K/S525) value, indicating a lower formation of metmyoglobin. As shown in Table 2, the (K/S572)/(K/S525) ratio gradually decreased with retail display time regardless of the treatments, suggesting the formation of metmyoglobin on the surface of lamb loin. Significantly (*p* < 0.05) higher (K/S572)/(K/S525) values were observed on lamb meat treated with glutamate and NADH compared to those with testosterone before the retail display (0 day). No significant difference in the (K/S572)/(K/S525) ratio was seen between lamb meat treated with the four metabolites during display, but the values were lower than that of the control sample. Therefore, the application of these metabolites on the surface of lamb loin had no enhancing impact on the colour stability of lamb loin during retail display.

### 3.3. Changes in the Surface Colour of Lamb Meat during Retail Display

#### 3.3.1. Effect of Muscles (Loin vs. Chop) on the Instrumental Colour of Male and Female Lamb Meat

**Figure 4:**
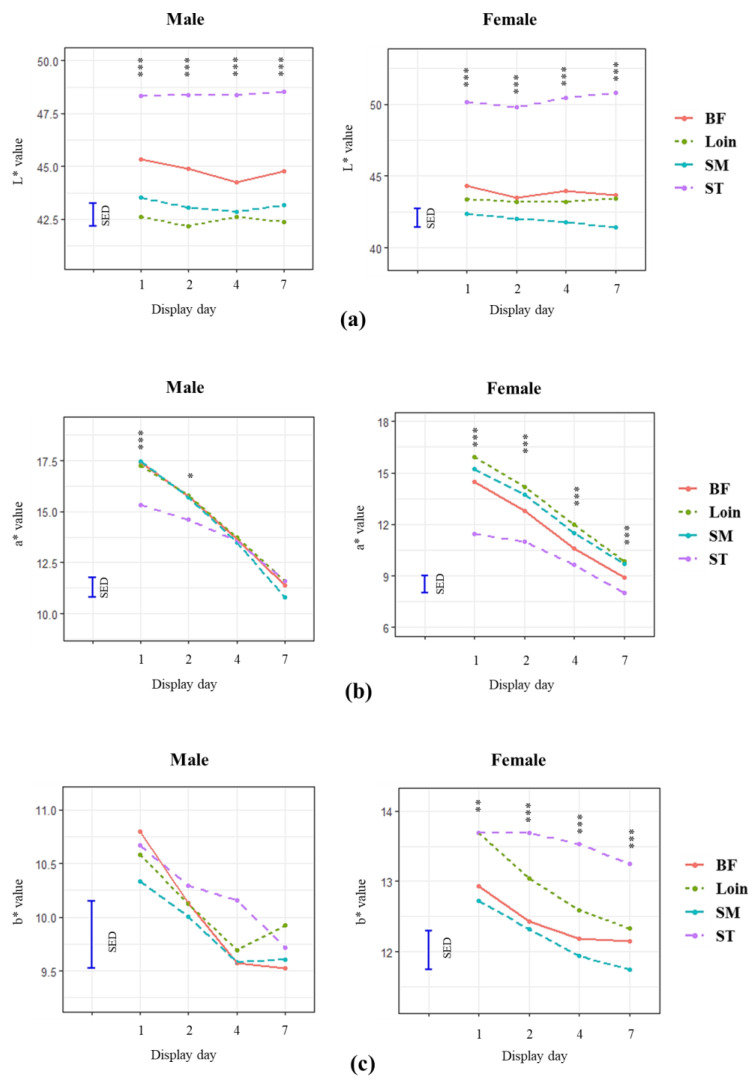

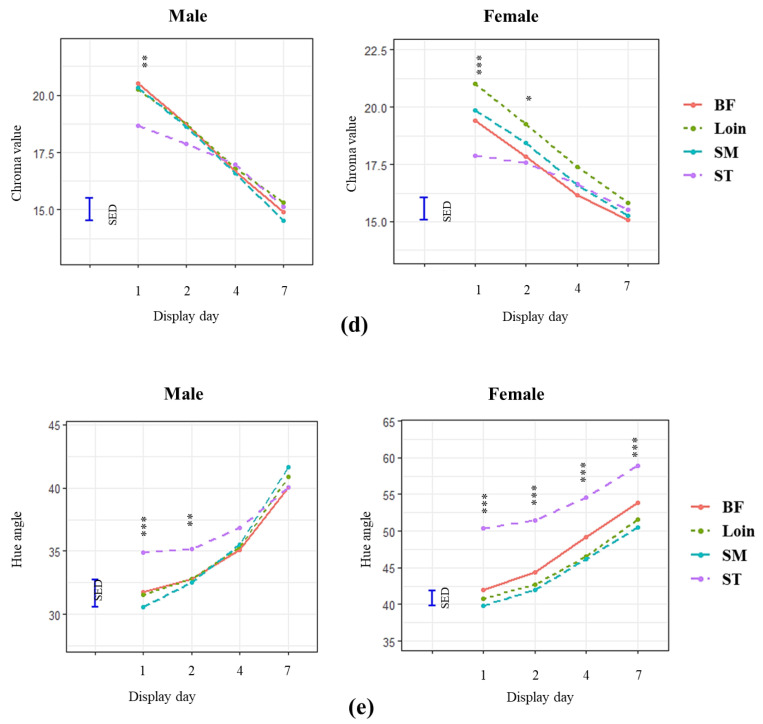
Effect of muscle types on colour properties (L* (**a**), a* (**b**), and b* (**c**), as well as chroma (**d**) and hue angle (**e**)) and colour stability of lamb from male and female animals during the simulated retail display for 1, 2, 4, and 7 days at 4 °C. SM = *m. semimembranosus*, BF = *m. biceps femoris*, and ST = *m. semitendinosus*. *p* < 0.0001 is presented as *** for the level of significance. *p* < 0.001 is presented as ** for the level of significance. *p* < 0.05 is presented as * for the level of significance. SED = standard error of the difference between mean values.

The surface colour of lamb loins from male and female animals during retail display of 7 days were compared to those from chop muscles, including SM, BF, and ST, and the results are shown in Figure 4.

Overall, a*, b*, and chroma decreased with display time with the increase in hue angle, as expected. There was no significant change observed in L* over the display period regardless of the muscles, although the ST muscle was lighter (higher L*, *p* < 0.05) than SM, BF, and loin. Significantly lower a* and chroma were observed in ST within 2 days of the display, and then the values became the same as SM, BF, and loin. For the male lamb, similar b* and hue were observed between the muscles, except for the higher (*p* < 0.001) hue angles that were seen in ST within the first 2 days of display. The ST from the female lamb had significantly higher b* and hue and lower a* than other muscles, while the difference reduced (*p* < 0.0001) with display time, indicating faster discolouration in SM, BF, and loin. The discolouration rate differed between sexes and muscles, suggesting that the colour stability of lamb meat may be affected by the interaction of sexes and muscles.

#### 3.3.2. Effect of Muscles on the Instrumental Colour of Lamb Leg Chops from Different Sexes and Colour Liable or Stable Sires

**Figure 5:**
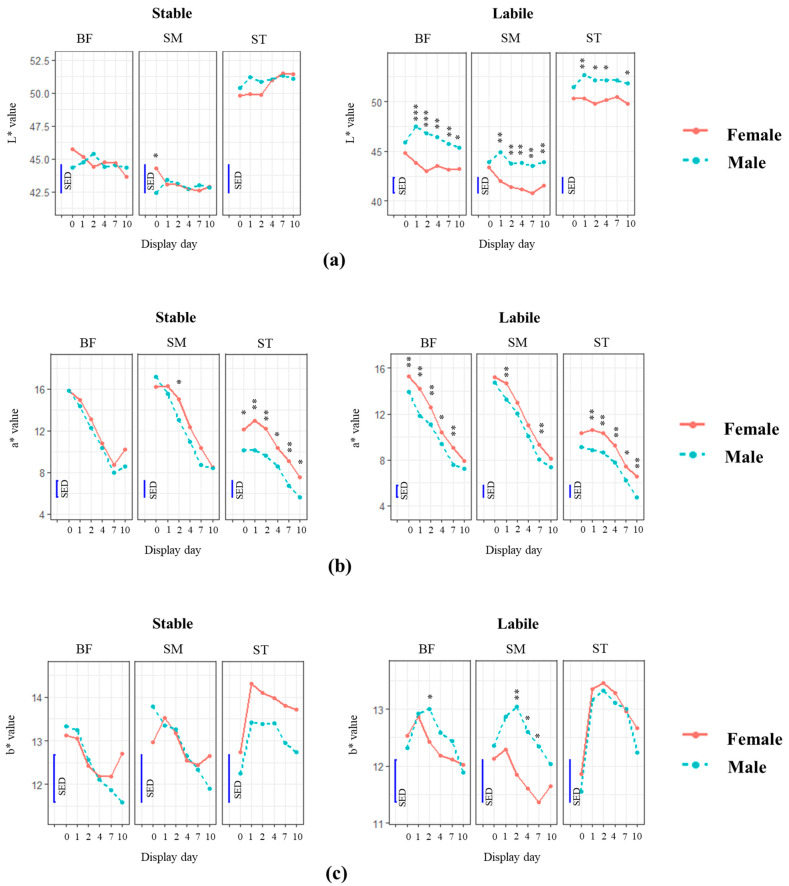

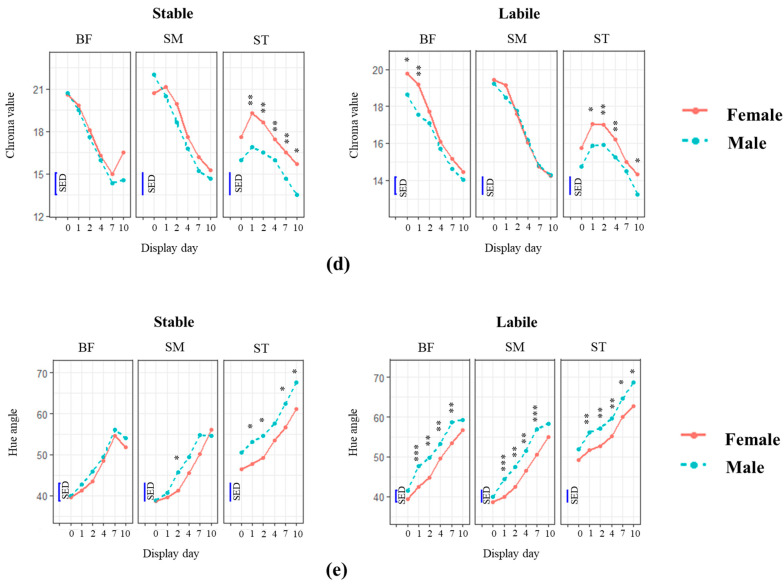
Effect of sex on colour properties (L* (**a**), a* (**b**), and b* (**c**), as well as chroma (**d**) and hue angle (**e**)) and colour stability of lamb chops from colour-stable and colour-labile sires during the simulated retail display for 1, 2, 4, 7, and 10 days at 4 °C. SM = *m. semimembranosus*, BF = *m. biceps femoris*, and ST = *m. semitendinosus*. *p* < 0.0001 is presented as *** for level of significance. *p* < 0.001 is presented as ** for level of significance. *p* < 0.05 is presented as * for level of significance. SED = standard error of the difference between mean values.

With the display time of up to 10 days, a*, b*, and chroma of lamb chop decreased regardless of muscles, sexes, and sires, while the brownness (hue angle) increased with display time (Figure 5). No significant changes in the display time were detected for L*, which is in line with the findings in the previous section in Section 3.3.1. ST had significantly higher L* and hue values than SM and BF throughout the display period of 10 days regardless of sexes and sires. The BF muscle had a lighter colour than SM, especially in lambs from the labile sire, suggesting the possible interaction between sire and muscle type on the colour stability of lamb meat.

The changes in a*, b*, and chroma between muscles differed depending on the sexes and sires. Overall, SM and BF muscles had higher a* and chroma with lower b* than ST during the display. The a* and chroma values of SM and BF muscles decreased faster than those of ST and reached a similar level to the values of ST after 2 days of display, while the a* value of the ST muscle remained lower (*p* < 0.05) than those of SM and BF except for the samples from female–stable lamb (from 4–10 days, *p* > 0.05). The b* of different muscles was similar before display and increased after storage for 1 day, especially on the ST muscle, and then gradually decreased with display time. ST had a higher yellowness than SM and BF; this difference was only significant in the meat from female animals.

#### 3.3.3. Effect of Sexes and Sires on the Instrumental Colour of Lamb Leg Chops

Overall, the pH values were similar between the sex and sire groups (*p* > 0.05). Lamb chops from male animals had lighter (higher L*), browner (higher hue angle), and less red colour (lower a* and chroma) than the female animals, with the difference being more significant (*p* < 0.05) in the ST muscle and meat from the labile sire (Figure 5). No significant difference in instrumental colour was observed in SM and BF muscles between male and female animals from stable sires (*p* > 0.05).

The colour properties of lamb leg chops from different sires are shown in Supplementary Figure S1. In general, lamb chops from stable sire had brighter red (higher a* and chroma) and less brown (lower hue angle) colour than those from the labile sire. Based on a*, b*, and chroma values, faster discolouration was also observed in SM and BF muscles from male–stable animals than in the other treatment combinations.

## 4. Discussion

Colour properties and stability during storage have been associated with the complex interplay of intrinsic (e.g., genetics, age, and muscles) and extrinsic factors (e.g., slaughtering, further processing, and packaging) [7,9,21]. Five experiments were carried out in this study to holistically investigate the effect of metabolites (NADH, glutamate, methionine, and testosterone), sexes, and sires on the colour properties and stability of lamb meat from loin and chops. The current study confirmed the previous findings by Subbaraj, Kim, Fraser, and Farouk [12] indicating that NADH and glutamate improved the colour stability, while methionine and testosterone had detrimental impacts on the colour stability of lamb meat.

**Figure 6:**
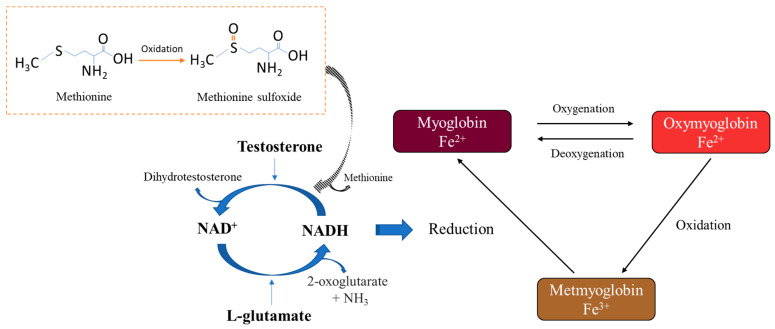
Possible mechanisms of the effect of metabolites (NADH, glutamate, methionine, and testosterone) on the metmyoglobin reductase activity in meat.

NADH is known for its essential role as an electron carrier to initiate the enzymatic and non-enzymatic reduction in metmyoglobin in meat [15,22,23], and the increasing levels of NADH improved the activity of metmyoglobin reductase (Table 1). The addition of glutamate, methionine, and testosterone into the reaction matrix played different roles in the reducing activities of metmyoglobin with NADH. The possible mechanisms of these metabolites on the reductase activity of metmyoglobin are shown in Figure 6. Metmyoglobin can be reduced by NADH to myoglobin, resulting in the bright red colour of oxymyoglobin after 5 min of incubation (Figure 2, control). NADH is oxidised and transformed into NAD^+^, which may react with glutamate to form NADH through the glutamate dehydrogenase pathway [24]. This may explain the positive impact of glutamate on the metmyoglobin reductase activity, as shown in Table 1. To the best of our knowledge, the mechanism of the negative impact of methionine on metmyoglobin reductase activity has not been studied. The possible biochemical pathway may involve the oxidation of methionine to its sulfoxide and reaction with NADH to form methionine through the methionine sulfoxide reductase pathway [25]. For the addition of testosterone, a significant reduction in metmyoglobin reductase activity was observed, which could be due to the reaction of testosterone with NADH and conversion to dihydrotestosterone through the 5α-reductase pathway [26]. The concentration of NADH in the reaction mixture was thus reduced, which consequently decreased metmyoglobin reductase activity (Table 1). Furthermore, the increased level of testosterone may limit the reducing activity of NADH with metmyoglobin and result in a delayed colour change from brown to a bright red colour, as shown in Figure 2 (testosterone, 2.0–8.0 mM). Such a detrimental effect of testosterone on the colour stability of lamb meat was further confirmed by the reduced activity of metmyoglobin reductase from male animals over the females (Figure 3).

The increase in metabolite concentration on the meat surface had no impact on the colour properties and stability of lamb loins, probably due to the biochemical reactions mainly occurring inside myocytes, instead of the external surface of the meat. Inherent alteration in metabolite content can be achieved by manipulating processing (e.g., production systems) and animal factors. Animal factors including breed [7], sexes [27,28], sire [12,29], and muscle types [9], have been suggested to affect the colour properties and stability of lamb meat. In this study, lamb meat from different muscles had different colour properties and levels of stability during retail display, which is likely associated with the fibre types of the muscles [30,31]. The colour stability of the ST muscle was better, with less degree of discolouration observed during simulated retail display than loin, SM, and BF muscles (Figure 4). This could be due to the higher glycolytic fibre (type IIB) content in ST muscle compared to SM, BF, and loin [30,32]. The colour properties of muscles with more glycolytic fibres (type IIB) are more stable than those with oxidative fibres (type I) [9]. The colour properties and stability of lamb meat from loins, SM, and BF were similar regardless of sex, while their surface colour deteriorated faster than ST muscle, especially in rams, suggesting the interaction between muscles and sexes [33]. Lamb meat from male animals generally had lighter, less red, and browner colour (higher L* and hue angle and lower a*, b*, and chroma) than those from females. Similar findings were also reported in previous studies indicating that lamb meat from male animals had higher L* [27,28,34] and lower a* and chroma values than those from the females [35,36,37]. Such variations in colour properties may be associated with different proportions of muscle fibre types between rams and ewes. Male animals have been reported to develop more glycolytic fibres (type II) than females [38], which may be associated with a higher level of testosterone in males [38,39].

Furthermore, the differences in colour properties between sexes were mainly found in lamb meat from the labile sire, suggesting that the sire groups of lamb may be another factor (Figure 5). The surface colour of chop muscles (ST, SM, and BF) from the stable sire group was bright red and less brown than those from the labile group, which may be associated with the impact of endogenous metabolites observed in this study. Previous studies have reported a higher level of NADH and glutamate and a lower level of testosterone and methionine in the colour-stable sire than in the labile group [12]. The possible metabolic pathways for the inherent variation in metabolite contents involve the metabolism of aminosugars (nucleosides and sugar phosphates) and amino acids. In this study, the colour properties of ST muscle from female–stable lambs were more stable (lower extent of discolouration) than other sex–sire combinations, suggesting the possible interaction among sex, sire, and muscle type, which warrants future work.

## 5. Conclusions

In this study, we performed five experiments to provide a holistic analysis of the effects of metabolites (NADH, glutamate, methionine, and testosterone), sex, sire, and muscle type on chilled lamb meat colour. The outcomes of this study confirmed the possible heritability of lamb meat colour and the positive effect of NADH and glutamate on the oxidative stability of colour, which may be associated with the glutamate dehydrogenase pathway. The negative impacts of testosterone and methionine on lamb meat colour may be attributed to their reactions with NADH, which results in lower levels of NADH in meat, thereby inhibiting the reduction in metmyoglobin. Future research should be carried out to validate the proposed mechanisms for the effect of metabolites on metmyoglobin reductase activity.

In addition, lamb meat colour and its stability during retail display were affected by sex, sire, and muscle types, and their interactions. The sex effect mainly caused changes in the colour properties of lamb meat (i.e., similar in the discolouration rate but differed in appearance). The sire effect also altered the initial colour of lamb meat, while more importantly, it influenced the oxidative stability of meat colour during retail display. The effects of sex on the properties of lamb meat colour were more pronounced in the lamb from the labile sire, suggesting the possible interactions between sex and sire. Muscle type is another key variant that interacts with sex and sire and influences both properties and oxidative stability of lamb meat colour.

## 6. Implications

The implications of this study for sustainable food systems include the following connotations:

(1) It is important to develop innovative production (e.g., different feeds) and processing strategies to enhance the beneficial metabolites (NADH and glutamate) in lamb meat for improved colour stability;

(2) Farmers should consider selecting colour-stable sires for breeding and produce lamb meat with a more stable colour during shelf storage to sustain the supply of high-quality meat for consumers;

(3) Utilising information about the sex and sire of lamb meat can help the meat industry to develop different marketing strategies to realise the value of different lamb meat and develop packaging technologies (e.g., active packaging) to maximise its shelf life;

(4) Consequently, the outcomes of this study may help the meat industry reduce meat waste and improve the sustainability of meat production practices.

## Figures and Tables

**Table 1:** Reduction in lamb metmyoglobin (MetMb) using lamb metmyoglobin extracts in various reaction mixtures at 30 °C and pH 6.4.

K4Fe(CN)_6_	EDTA	MetMb(III)	MetMb Extract	NADH	Glutamate	Methionine	Testosterone	Activity (nmol·min^−1^·g^−1^)
+	+	+	+					0
+	+	+	+		+			−2.0
+	+	+	+			+		6.2
+	+	+	+				+	−1.8
+	+	+	+	+				337.8
+	+	+	+	+	+			368.5
+	+	+	+	+		+		317.3
+	+	+	+	+			+	133.4

“+” This indicates the compounds that were added to the reaction mixture.

**Table 2:** Effects of metabolites on the formation of metmyoglobin on muscle surfaces of lamb loins evaluated with reflectance measurements (mean ± SD).

	^#^ (K/S)_572_/(K/S)_525_				
Display Day	0 d	1 d	2 d	4 d	7 d
Control	1.735 ± 0.067 ^ab^	1.518 ± 0.078	1.355 ± 0.077 ^a^	1.173 ± 0.077 ^a^	1.002 ± 0.118 ^a^
Glutamate	1.763 ± 0.093 ^a^	1.520 ± 0.073	1.340 ± 0.074 ^ab^	1.134 ± 0.083 ^b^	0.955 ± 0.154 ^b^
Methionine	1.731 ± 0.063 ^ab^	1.502 ± 0.068	1.320 ± 0.066 ^ab^	1.121 ± 0.080 ^b^	0.935 ± 0.114 ^b^
NADH	1.762 ± 0.137 ^a^	1.517 ± 0.073	1.316 ± 0.080 ^b^	1.107 ± 0.089 ^b^	0.942 ± 0.127 ^b^
Testosterone	1.709 ± 0.092 ^b^	1.506 ± 0.065	1.332 ± 0.069 ^ab^	1.134 ± 0.075 ^b^	0.965 ± 0.122 ^ab^

^#^ (K/S)_572_/(K/S)_525_ was calculated as the ratio of reflectance at 572 nm and 525 nm. Different letters of “a or b” within the same column mean results are significantly different from each other (*p* < 0.05).

## Data Availability

Data is contained within the article or Supplementary Material.

## Supplementary Materials

The following supporting information can be downloaded at: https://www.mdpi.com/article/10.3390/foods12214031/s1, Figure S1: Effect of sire on colour properties and colour stability of lamb chops from male and female animals during the simulated retail display for 1, 2, 4, 7, and 10 days at 4 °C. BF = *m. biceps femoris*, SM = *m. semimembranosus*, and ST = *m. semitendinosus*. *p* < 0.0001 is presented as *** for the level of significance. *p* < 0.001 is presented as ** for the level of significance. *p* < 0.05 is presented as * for the level of significance. SED = standard error of the difference between mean values.
