# Supplementary material for: Effects of Metabolites, Sex, Sire, and Muscle Type on Chilled Lamb Meat Colour
Source: Foods. 2023 Nov 5;12(21):4031. doi: 10.3390/foods12214031 (PMC10650127; doi:10.3390/foods12214031)
Supplement: Supplementary file 1 [file foods-12-04031-s001.zip › foods-2596971-supplementary.pdf]

Effects of metabolites, sex, sire, and muscle type on chilled lamb meat colour

Renyu Zhang [1], Guojie Wu [1], Maryann Staincliffe [2], John C. McEwan [3], and
Mustafa M. Farouk [1*]

[1] Food Technology & Processing Team, AgResearch Ltd, Palmerston North, New Zealand

[2] Statistics Team, AgResearch Ltd, Hamilton, New Zealand

[3] Animal Genomics Team, AgResearch Ltd, Puddle Alley, Mosgiel, New Zealand

[*]Corresponding author e-mail: mustafa.farouk@agresearch.co.nz

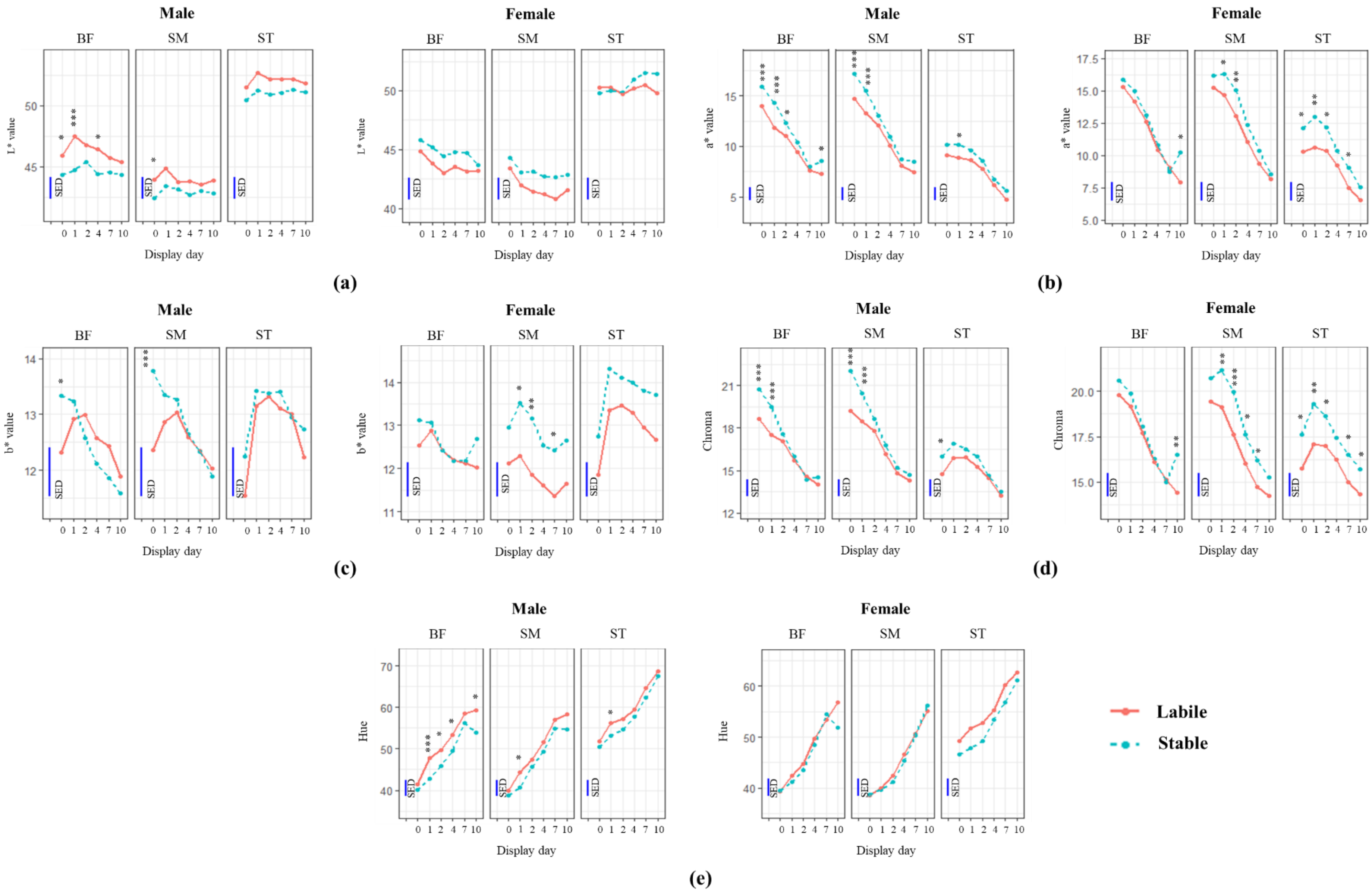

Male
Female
BF
SM
ST
L* value
a* value
b* value
Chroma
Hue
Display day
SED
(a)
(b)
(c)
(d)
(e)
Labile
Stable

Figure S1: Effect of sire on colour properties (L* (**a**), a* (**b**), and b* (**c**), as well as chroma (**d**) and hue angle (**e**)) and colour stability of lamb chops from male and female animals during the simulated retail display for 1, 2, 4, 7, and 10 days at 4 °C. BF = *m. biceps femoris*, SM = *m. semimembranosus*, and ST = *m. semitendinosus*. $p < 0.0001$ is presented as *** for the level of significance. $p < 0.001$ is presented as ** for the level of significance. $p < 0.05$ is presented as * for the level of significance. SED = standard error of the difference between mean values.
